# Biosystematics and Taxonomic Treatment of Three New *Kengyilia* Species in the Triticeae (Poaceae)

**DOI:** 10.1002/ece3.74040

**Published:** 2026-07-23

**Authors:** Xiao‐Yang Pan, Shuang‐Dan Li, Chang‐Bing Zhang, Jia‐Yue He, Yuan‐Yuan Lu, Ting‐Ting Zheng, Ying‐Hui Li, Li‐Li Xu, Hai‐Qin Zhang, Hou‐Yang Kang, Dan‐Dan Wu, Yong‐Hong Zhou

**Affiliations:** ^1^ State Key Laboratory of Crop Gene Exploration and Utilization in Southwest China Sichuan Agricultural University Chengdu Sichuan China; ^2^ Triticeae Research Institute Sichuan Agricultural University Chengdu Sichuan China; ^3^ Sichuan Academy of Grassland Science Chengdu China; ^4^ College of Grassland Science and Technology Sichuan Agricultural University Chengdu Sichuan China

**Keywords:** genome, *Kengyilia*, morphology, new species, phylogenetic relationship, StYP

## Abstract

In the present study, we discovered and investigated three new species of *Kengyilia* (Triticeae, Poaceae): *Kengyilia changduensis* C. B. Zhang, H. Q. Zhang et Y. H. Zhou, *Kengyilia dingqinensis* Y. H. Zhou, D. D. Wu et X. Y. Pan, and *Kengyilia tibetica* Y. H. Zhou, C. B. Zhang et D. D. Wu, in Changdu, Tibet, China based on morphological, cytological, and molecular data. Each species is morphologically characterized and distinguished from existing *Kengyilia* species by smooth internodes, glumes oblong‐ lanceolate and awns 2 mm, lemmas pubescent and awns 5–10 mm of *K. changduensis*, the lower part of the spikes is sparse, internodes hairy, glumes covered with soft hairs, awns 2 mm, lemmas oblong and awns 5 mm of *K. dingqinensis*, and internodes hairy, glumes oblong‐lanceolate and awns 2–6 mm of *K. tibetica*. The genomic in situ hybridization results confirm that *K. changduensis*, *K. dingqinensis*, and *K. tibetica* are allohexaploid with StStYYPP genomic constitutions. Phylogenetic analyses, based on *GBSSI* nuclear gene and chloroplast genome sequences, suggest that *K. changduensis*, *K. dingqinensis*, and *K. tibetica* were closely related to genera *Pseudoroegneria*, *Roegneria* and *Agropyron*, and the *Roegneria* served as the maternal donors during its polyploid speciation of *K. changduensis* and *K. tibetica*, and *Agropyron* served as the maternal donors of *K. dingqinensis*.

## Graphical abstract

Three new *Kengyilia* species have been discovered. These new species have morphological differences from their closely related species. Three *Kengyilia* species are allohexaploid and contain the StStYYPP genome. Different *Kengyilia* have different origins.

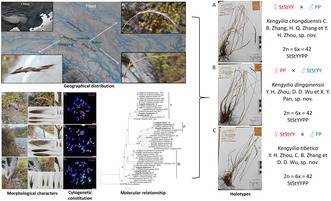

## 1 Introduction

The genus *Kengyilia* C. Yen et J. L. Yang (Triticeae, Poaceae) is an independent allohexaploid group established by Yen & Yang with *Kengyilia gobicola* C. Yen et J. L. Yang as the type species (Yen and Yang 1990). Currently, *Kengyilia* comprises 26 species and six varieties, distributed primarily in Central Asia and the Qinghai–Tibetan Plateau. Species of *Kengyilia* exhibit several common morphological characteristics, for example, lemmas covered with hairs, glumes with obvious ridges, and underdeveloped apical spikelets (Yen and Yang 2013). Species of the genus thrive in diverse habitats, including subalpine meadows, alpine meadows, mountain grasslands, desert steppes, and Gobi deserts, at elevations ranging from 1100 to 5200 m (Yang et al. 1992; Cai and Zhi 1999; Yen and Yang 2013). *Kengyilia* is an important component of the plateau grassland ecosystem.

Cytogenetic studies have demonstrated that all currently recognized species of *Kengyilia* are allohexaploids possessing the genomic constitution StStYYPP, in which the StStYY genomes and PP genome were dominated by the genus *Roegneria* and *Agropyron*, respectively (Löve 1984; Wang et al. 1994; Yen and Yang 2013). Genomic in situ hybridization (GISH) is a powerful method to determine the genomic composition and intergenomic rearrangements of polyploid species (Ørgaad and Heslop‐Harrison 1994; Zhang et al. 2006). In species with uncertain genomic constitutions, GISH can be used to infer chromosomal homology by hybridizing genomic DNA from putative progenitor taxa to target chromosomes. Tan et al. (2021) discovered that the genome composition of *Campeiostachys purpuraristata* (C. P. Wang & H. L. Yang) Y. H. Zhou, H. Q. Zhang et W. H. Chen and *C. calcicola* (Keng) Y. H. Zhou, H. Q. Zhang et M. Q. Deng is StStYYHH, and a translocation phenomenon between the Y and H genomes in *C. purpuraristata* was identified. Nevertheless, the diploid donors of several Triticeae genome are unavailable for some taxa. Therefore, GISH alone is insufficient for the full identification of the genomic compositions of all allopolyploid species, particularly those allotetraploids containing unknown genomes such as Y and Xm.

Single‐ or low‐copy nuclear genes provide an effective alternative for elucidating the origins of polyploid species. Nuclear gene, such as granule‐bound starch synthase I (*GBSSI*), have been widely used to reconstruct the evolutionary history of polyploid taxa (Popp et al. 2005; Tan et al. 2021, 2022; Sha et al. 2024). For example, Sha et al. (2024) verified the genomic composition of a newly described species, *Roegneria yenchiana* X. Fan et L. N. Sha, based on phylogenetic analyses of single‐copy nuclear genes. In addition, chloroplast sequences have also been extensively utilized to infer maternal ancestry and modes of hybrid speciation in polyploid plants because chloroplast genomes are typically maternally inherited in angiosperms (Palmer 1985; Chen et al. 2020). Compared with nuclear genomes, plastomes are highly conserved in structure and gene content, largely free from recombination, and possess a shorter coalescent time at approximately half the speed of nuclear regions (Birky et al. 1983; Palmer 1985; Wicke et al. 2011; Walker et al. 2014). These advantageous features make the chloroplast sequences particularly valuable for reconstructing phylogenetic relationships and tracing evolutionary histories (Jansen et al. 2008; Walker et al. 2014; Chen et al. 2020; Chen, Sha, et al. 2021). Using nuclear ribosomal internal transcribed spacer (ITS) sequences, Liu et al. (2006) demonstrated that *Pseudoroegneria* (St) is the donor of the St genome in *Kengyilia*. Subsequently, Zhang et al. (2009) speculated that *Agropyron* (P) serve as the maternal donor of *Kengyilia melanthera* (Keng) J. L. Yang, C. Yen et B. R. Baum, *K. mutica* (Keng) J. L. Yang, C. Yen et B. R. Baum and *K. thoroldiana* (Oliver) J. L. Yang, C. Yen et B. R. Baum, and the maternal donor of other *Kengyilia* species in the research was St or Y genome in *Roegneria*. Based on an analysis of chloroplast genome sequences, Chen, Yan, et al. (2021) identified that *Pseudoroegneria* was the maternal donor of perennial species, including most *Kengyilia* and other St‐genome containing polyploids. Consequently, the combined analysis of single‐ or low‐copy nuclear genes and chloroplast sequences provides a powerful framework for reconstructing the evolutionary history and genomic origins of polyploid species.

In 2023, we discovered three populations in Tibet (China), that morphologically resemble *Kengyilia* based on single spikelet per node, compact spikes, and pubescent lemmas. However, these populations differed from all previously described *Kengyilia* species in several stable diagnostic characters, including the length of lemma and glume awns and the presence of pubescence glumes and lemmas. We therefore recognized them as three previously undescribed species and temporarily designated them as *Kengyilia changduensis* C. B. Zhang, H. Q. Zhang et Y. H. Zhou, *Kengyilia dingqinensis* Y. H. Zhou, D. D. Wu et X. Y. Pan, and *Kengyilia tibetica* Y. H. Zhou, C. B. Zhang et D. D. Wu, respectively. These species occur in the Hengduan Mountain Region, especially along the Zhaqu and Sequ river valleys at elevations of 3500–3800 m. To evaluate their taxonomic status, we investigated their morphology, genomic constitution, and phylogenetic relationships using GISH, nuclear *GBSS1* sequences, and complete chloroplast genome data. The objectives of this study were to: (1) characterize the morphological variation of the three new species; (2) determine their genomic constitution using GISH; (3) infer their phylogenetic relationships with related Triticeae taxa using nuclear and plastome data; and (4) provide formal taxonomic descriptions and nomenclature treatment.

## 2 Materials and Methods

### 2.1 Plant Sampling

**FIGURE 1:**
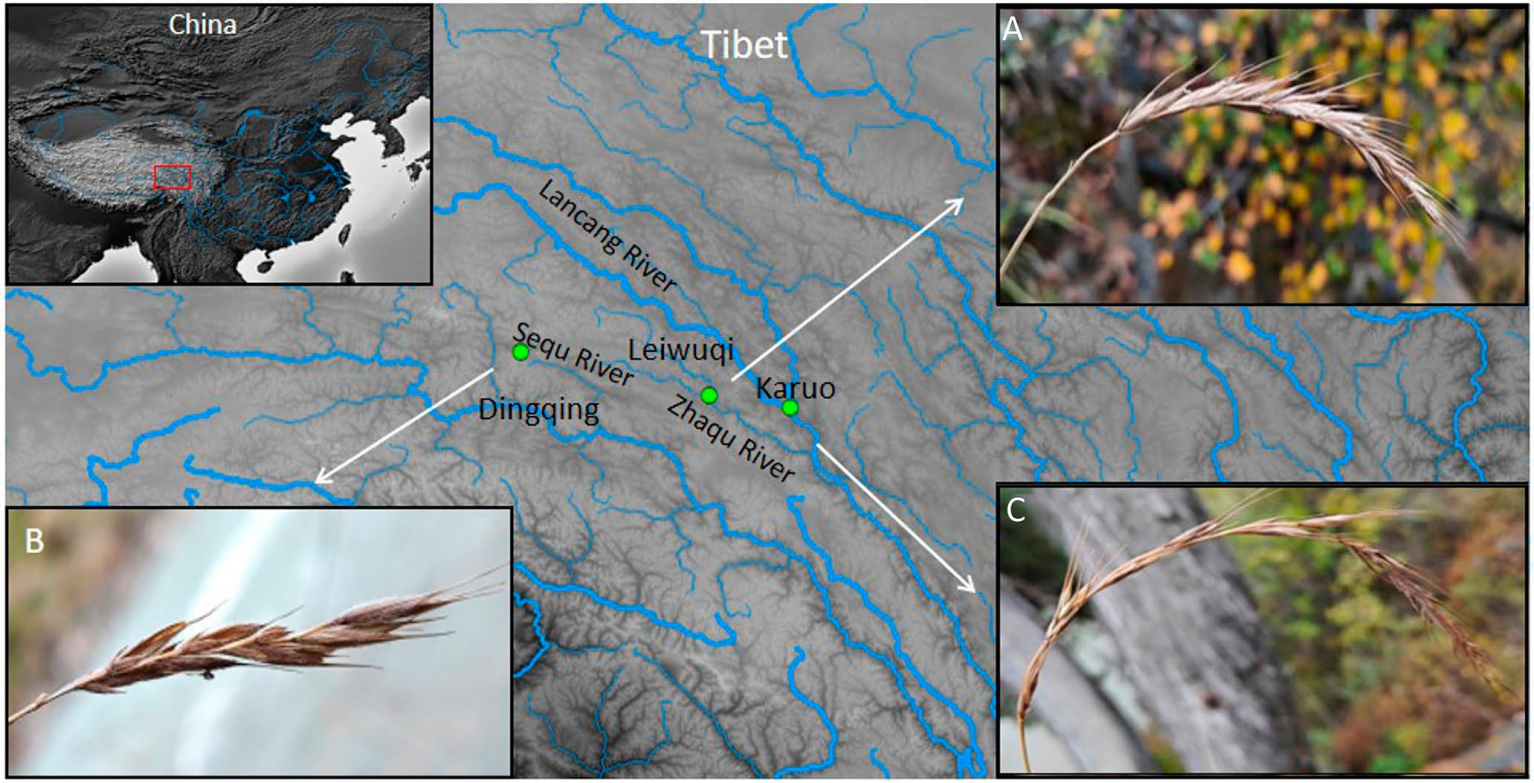
Location of the three new *Kengyilia* species samples. (A) *Kengyilia changduensis*. (B) *K. dingqinensis*. (C) *K. tibetica*.

Seeds and vegetative parts of three putative new species were collected from Changdu, Tibet, China. We found those plants were restrictedly distributed in Leiwuqi, Dingqing, and Karuo county (Figure 1). For each species, at least three individuals were sampled. Voucher specimens were prepared from fruiting individuals and include pressed whole plants, inflorescences, and detached spikelets. All vouchers were deposited at the Herbarium of Triticeae Research Institute, Sichuan Agricultural University, China (SAUTI) under the following accession numbers: 24925‐2, 24925‐7, and 24925‐12, corresponding to *K. changduensis*, *K. dingqinensis*, and *K. tibetica*.

### 2.2 Morphological Observation

Morphological features were recorded for the following structures: underground rhizome, stalk, cauline internode, leaf auricle, leaf blade, spike orientation and density, spikelet arrangement, rachis, glume shape and awn, lemma shape and awn, palea apex, and anther color and length. At least 20 independent measurements per quantitative trait were taken from fresh material using a stereomicroscope (Olympus SZX7, Tokyo, Japan) with a digital camera. Principal components analysis (PCA) was performed on the correlation matrix of log‐transformed morphological variables using the stats package in R v4.5.1.

### 2.3 Genomic In Situ Hybridization (GISH)

Roots tip with meristems were collected from germinating seeds and actively growing adult plants, prefixed with nitrous oxide at 0.1 MPa for 2.5 h, and fixed in 90% glacial acetic acid for 5 min. Chromosome preparations were performed using drop methods as described by Wu et al. (2025). To characterize the chromosome constitution of the natural hybrid, the whole genome DNAs of *Roegneria ciliaris* (2*n* = 4*x* = 28, StStYY), *Pseudoroegneria stipiflolia* (2*n* = 2*x* = 14, StSt), and *Agropyron cristatum* (2*n* = 2*x* = 14, PP) were separately isolated using cetyltrimethyl ammonium bromide (CTAB) procedure (Doyle and Doyle 1990). The gDNA (1000–1500 ng/μL) was then labeled with dUTP‐ATTO‐550 or dUTP‐ATTO‐488 using a nick translation labeling kit (Jena Bioscience, Jena, Germany) as multicolored GISH (mc‐GISH) probes. The 4′,6‐diamidino‐2‐phenylindole (DAPI) was used for slide counterstain. Images were captured with an Olympus BX51 fluorescence microscope equipped with a Photometric SenSys DP‐70 CCD camera (Olympus, Tokyo, Japan). Among them, blue fluorescence was excited by UV light exposure for 30 ms, red fluorescence by 535 nm light for 1 s, and green fluorescence by 488 nm light for 1.5 s.

### 2.4 DNA Amplification and Sequencing

The nuclear *GBSSI* sequences were amplified with primers and thermocycling conditions for amplification of genes listed in Tables S1 and S2. PCR amplification was carried out in a 25 μL reaction mixture, containing 10 × ExTaq polymerase buffer, 1.5 U ExTaq (TaKaRa, Dalian, China), 200 μM of dNTP, 1 μM of each primer, and about 50 ng of template DNA. Amplifications were performed on the Veriti 96‐Well Thermal Cycler (Thermo Fisher Scientific, USA). The PCR products were visualized on 1.0% agarose gels, purified by a FastPure Gel DNA Extraction Mini Kit (Vazyme, Nanjing, China), and then cloned into pMD19‐T vector (TaKaRa, Dalian, China) according to the manufacturer's instructions. We randomly selected a sample from each species for sequencing. To obtain all haplotypes for each material, at least 15 clones were randomly selected at once for sequencing until all haplotypes were identified. All clones were sequenced in both directions in Sangon Biotech (Shanghai) Co. Ltd. To prevent the inadvertent inclusion of error‐prone sequences, we selected a single representative for each gene fragment per allele of every accession—specifically, the unique sequence type that occurred most frequently among the cloned PCR products—and incorporated only one sequence of each haplotype into the matrix.

### 2.5 Plastome Genome Sequencing, Assembly, and Annotation

Short‐insert library (insert size, 300 bp) was prepared and then sequenced using the DNBSEQ‐T7 high‐throughput sequencing platform in Biomarker (Beijing, China). The complete genome was assembled using SPAdes v3.6.1 (Prjibelski et al. 2020). The plastome of *Triticum aestivum* (GenBank accession number NC002762) and *Hordeum vulgare* (GenBank No. EF115541) was used as the reference genomes for assembly of all new sequenced accessions. The putative plastid scaffolds were identified using the mapping algorithm and assembled in Bandage v0.8.1 (Wick et al. 2015). The genome was annotated using the online annotation tool CPGAVAS2 (https://47.96.249.172:16019/analyzer/annotate).

### 2.6 Phylogenetic Analysis

A total of 37 St‐genome‐containing polyploidy species and 39 diploid taxa representing 29 basic genomes in Triticeae were included in this study, and their *GBSSI* (Granule‐Bound Starch Synthase I) and chloroplast genomes DNA sequences for phylogenetic analyses were obtained from published data (Table S3). To confirm the relationship between the three new species and their close relatives, we conducted an analysis of single‐copy nuclear gene *GBSSI* and chloroplast genomes. Multiple sequence alignment was conducted using MAFFT v7.490 (Katoh and Standley 2013), with default parameters and additional manual edits to minimize gaps. Phylogenetic analyses were conducted using maximum likelihood (ML). ML analysis was performed using IQ‐TREE v2.0.7 (Minh et al. 2020). The stochastic models of nucleotide sequence changed due to different nucleotide substitution rates (Fan et al. 2013). The optimal model was TN + F + R3 for *GBSSI* data and TVM + I + G for chloroplast genome data. Maximum likelihood heuristic searches were performed with 100 random addition sequence replications and a Tree Bisection‐Reconnection (TBR) branch‐swapping algorithm. To infer the robustness of clades, bootstrap support (BS) values were calculated with 1000 replications. *Bromus catharticus* Vahl was designated as the outgroup for the GBSSI analysis, and *Brachypodium distachyon* (L.) P. Beauv. for the plastome analysis.

## 3 Results

### 3.1 Morphological Characters of Three Putative Newly *Kengyilia* Populations

**FIGURE 2:**
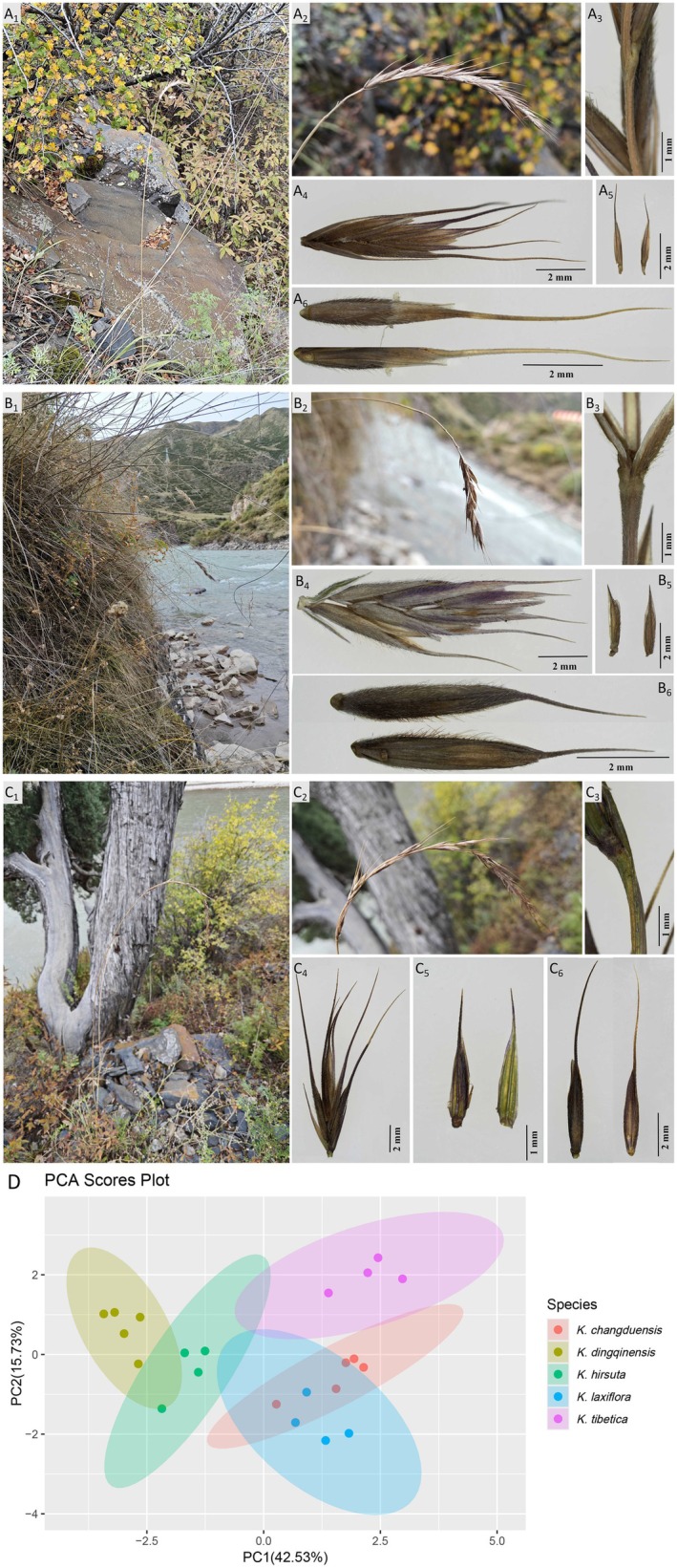
Morphological features of the three new species in *Kengyilia*. (A) *Kengyilia changduensis*; (B) *K. dingqinensis*; (C) *K. tibetica*; A_1_, B_1_, and C_1_: Habitat; A_2_, B_2_, and C_2_: Inflorescences; A_3_, B_3_, and C_3_: Internodes; A_4_, B_4_, and C_4_: Spikelets; A_5_, B_5_, and C_5_: Glumes; A_6_, B_6_, and C_6_: Lemmas and paleas; (D) Principal component analysis (PCA) of the *K. laxiflora* , *K. hirsute*, and three new species using morphological traits.

Morphologically, both *K. changduensis* and *K. tibetica* are similar to *Kengyilia laxiflora* (Keng) J. L. Yang, C. Yen et B. R. Baum, but differed by several diagnostic traits, most notably the longer awns on their lemmas and glumes. *K. tibetica* displayed pubescent internodes, whereas *K. changduensis* has entirely glabrous internodes. *K. dingqinensis* is morphologically similar to *Kengyilia hirsuta* (Keng) J. L. Yang, C. Yen et B. R. Baum, but is characterized by soft hairs on the internodes combined with awned and hairy glumes (Figure 2A–C). Principal component analysis (PCA) based on morphological dataset separated *K. laxiflora* , *K. hirsute*, and three putative species without overlap (Figure 2D). Geographically, *K. laxiflora* and *K. hirsuta* are distributed in the Hengduan Mountain region, and *K. laxiflora* has not been recorded from Changdu, Tibet (Yen and Yang 2013), which further supported the distinctiveness of these populations. Collectively, the morphological evidence, supported by the PCA and geographic considerations, demonstrated that the three populations represent distinct lineages. We therefore recognize the as new species: *Kengyilia changduensis* C. B. Zhang, H. Q. Zhang et Y. H. Zhou, *Kengyilia dingqinensis* Y. H. Zhou, D. D. Wu et X. Y. Pan, and *Kengyilia tibetica* Y. H. Zhou, C. B. Zhang et D. D. Wu.

### 3.2 Genome Composition of the Three New *Kengyilia* Species Using GISH

**FIGURE 3:**
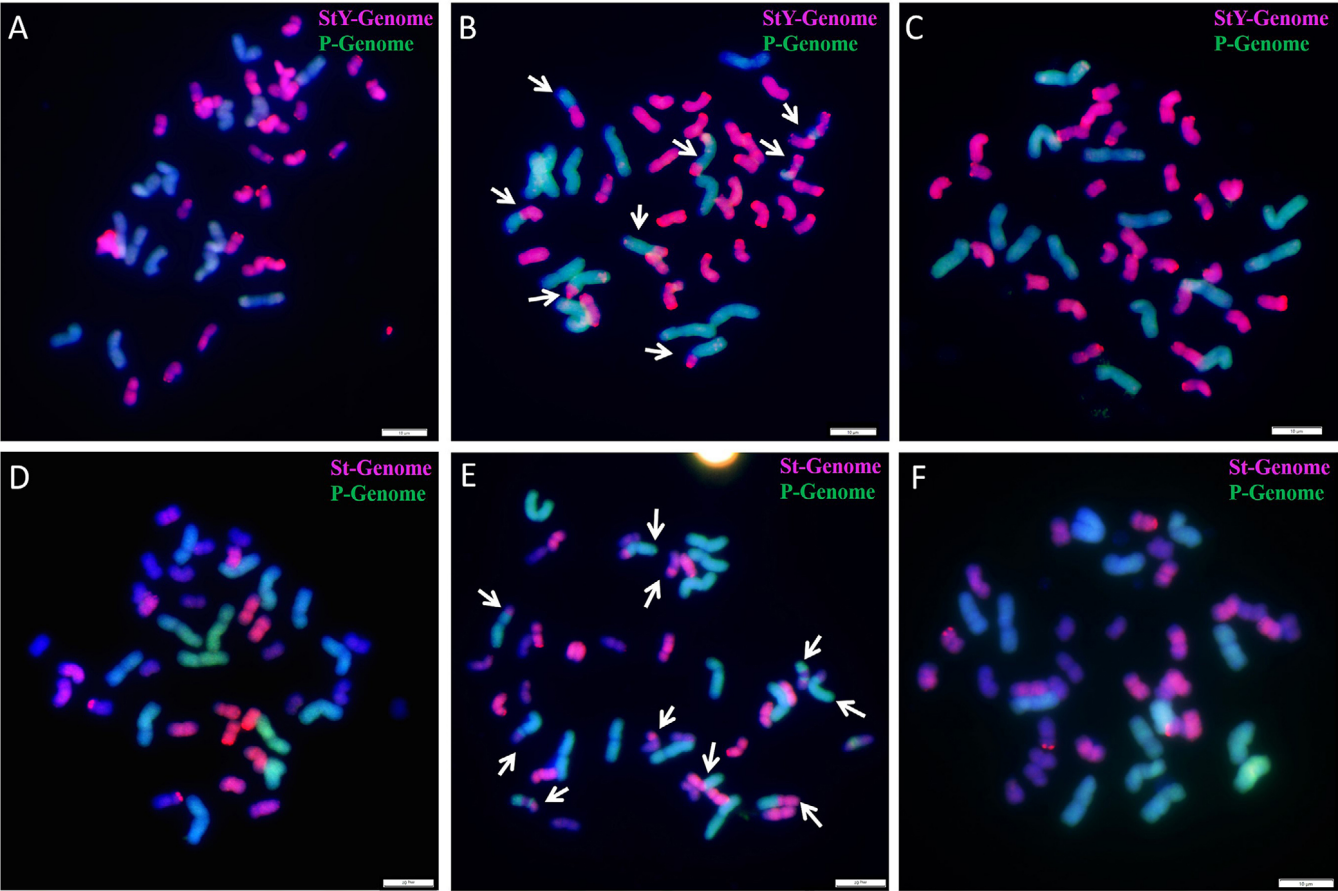
The genome constitution of three new *Kengyilia* species based on GISH. (A, D) *Kengyilia changduensis*; (B, E) *K. dingqinensis*; (C, F) *K. tibetica*. (A–C) GISH probes using the whole genomic DNA of *R. ciliaris* with StSYY genome in magenta and *A. cristatum* with PP genome in green fluorescence. (D–F) The DAPI in blue, whole genomic DNA of *P. stipiflolia* with StSt genome in magenta, and *A. cristatum* with PP genome in green fluorescence. White arrows indicates the chromosome structure variations. The scale bar equals 10 μm.

To determine the genome constitution of the three new species, GISH was carried out using the whole genomic DNA of tetraploid *Roegneria ciliaris* (Trin.) Nevski with StStYY genome, diploid *Pseudoroegneria stipifolia* (Czern. ex Nevski) Á. Löve with StSt genome, and diploid *Agropyron cristatum* (L.) Gaertn. with PP genome as probes. All three species contained 42 chromosomes, comprising 28 chromosomes with StStYY genome signals and 14 chromosomes with PP genome signals, confirming the StStYYPP allohexaploid constitution (Figure 3A–C). Notably, *K. dingqinensis* displayed four pairs of chromosomes showing intergenomic translocations between StY and P genome (Figure 3B). To clarify these chromosome translocations, multi‐colored GISH analysis was conducted using St and P genome probes. The results revealed that four chromosomal structural variations were involving one St/P chromosome pair and three Y/P chromosome pairs. In addition, we identified the fifth structural variation involving chromosome pair between the St/Y (Figure 3E). Accordingly, the genomic composition of *K. changduensis*, *K. dingqinensis*, and *K. tibetica* can be described as StStYYPP.

### 3.3 Phylogenetic Analyses of *GBSSI*

**FIGURE 4:**
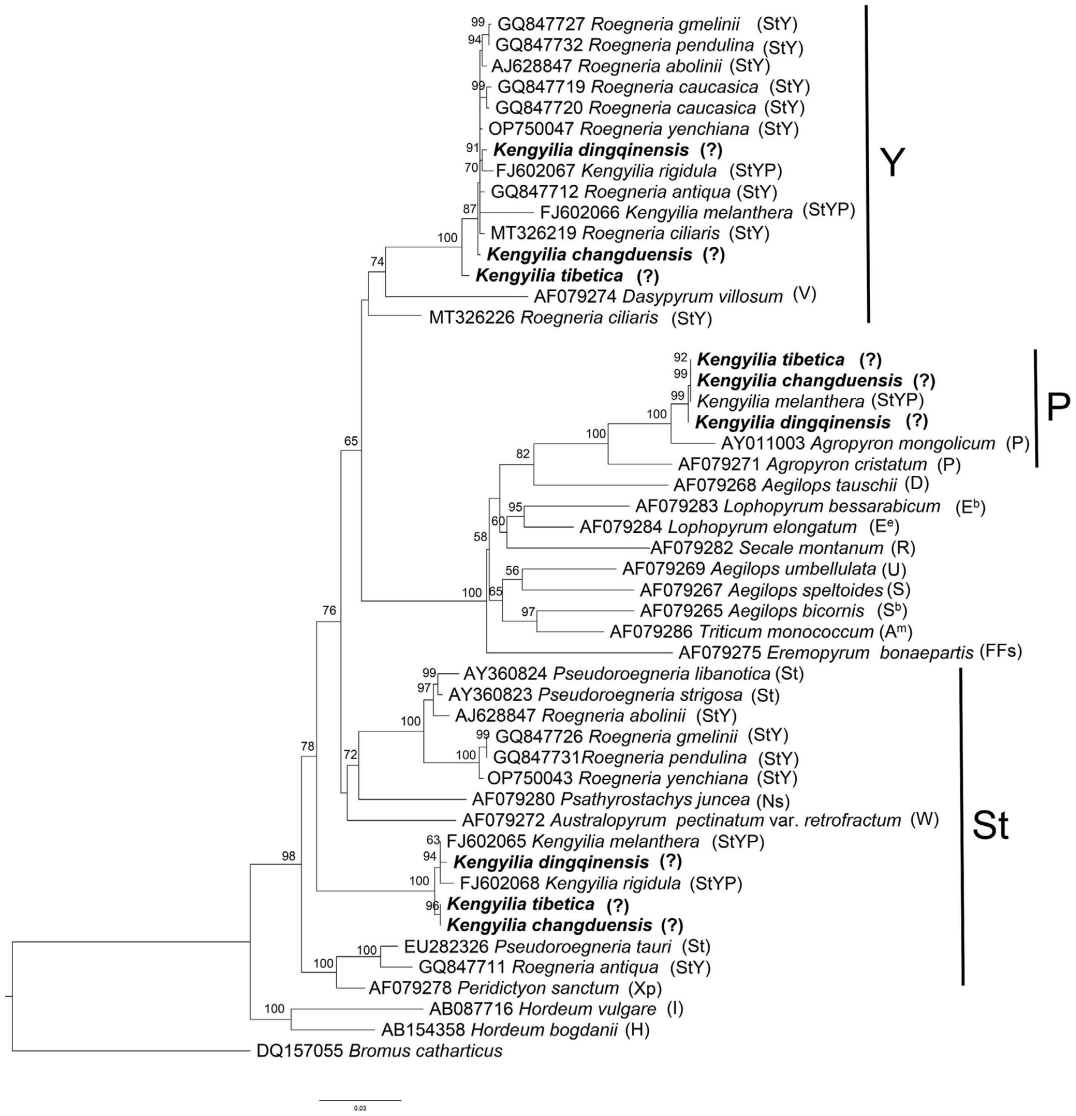
Maximum‐likelihood tree derived from the *GBSSI* dataset for *Kengyilia changduensis*, *K. dingqinensis*, *K. tibetica*, and related species. The numbers above the branches present bootstrap (BS) values > 50%. Upper case letters in parentheses indicate the genomic composition of each species, while the “?” signifies that the genomic composition is unknown.

The Maximum‐likelihood (ML) analysis of the *GBSSI* data from 35 species of Triceae yielded a single phylogenetic tree with Lnlikelihood value of 8857.651 (Figure 4). *Bromus cathartcus* Vahl. was used as an outgroup. The *GBSSI* sequences from *Kengyilia tibetica*, *K. changduensis*, and *K. dingqinensis* were categorized into three distinct clades corresponding to the St, Y, and P genomic types. The St‐type sequences from three new species clustered with St‐type sequences of other *Kengyilia* species and related genera, such as *Roegneria* with StStYY genome and *Pseudoroegneria* with StSt genome forming a paraphyletic grade. Similarly, the Y‐type sequences from three species grouped with those of *Roegneria* in a paraphyletic group. In contrast, the P‐type sequences from the three *Kengyilia* species formed a well‐supported monophyletic clade that was closely related to sequences of *Agropyron* species. Therefore, these phylogenetic patterns unambiguously demonstrated that *K. changduensis*, *K. dingqinensis*, and *K. tibetica* contained St, Y, and P subgenomes.

### 3.4 Genome Sequencing, Plastome Assembly, and Phylogenetic Analyses

**FIGURE 5:**
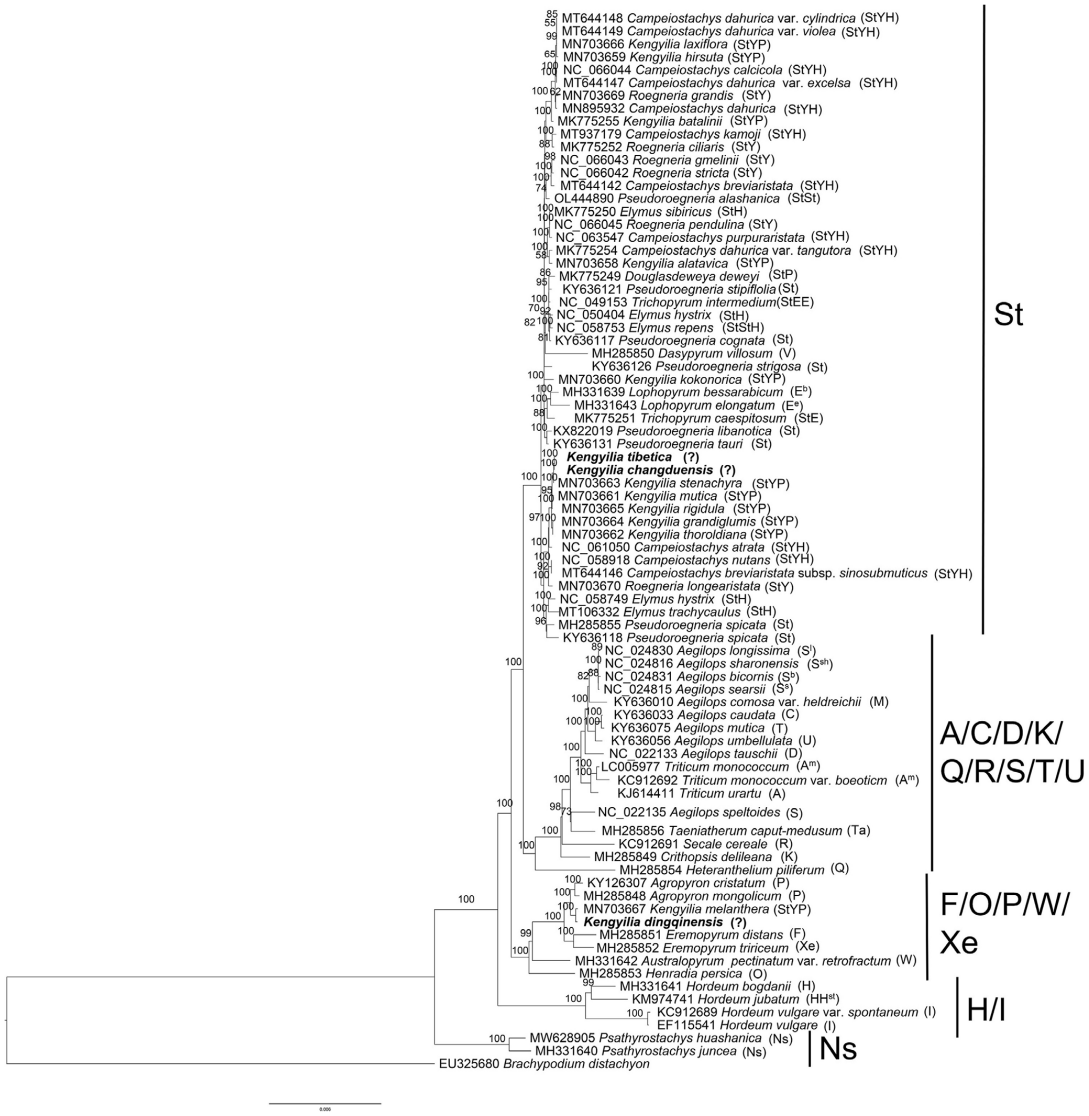
Maximum‐likelihood tree inferred using the complete chloroplast genome sequences for the 3 new species (*Kengyilia changduensis*, *K. dingqinensis*, *K. tibetica*) and their relatives in the Triticeae. The Numbers above nodes represent bootstrap values (BS ≥ 50%). The capital letters in brackets indicate the genome type of each species. The “?” symbol indicates that the unknown genomic composition of the species.

We sequenced, assembled and annotated the whole chloroplast genome of the *K. changduensis*, *K. dingqinensis*, *K. tibetica* (Figure S1). The lengths of the plastomes ranged from 135,176 to 135,663 bp. We conducted phylogenetic analysis using 76 whole plastome genome data sets of Triticeae species and *Brachypodium distachyon* (L.) P. Beauv. was used as outgroup, applying the TVM + I + G model (Lnlikelihood = 294,976.570) (Figure 5). The result indicated that the whole plastome sequences of these three new *Kengyilia* species and related species were split into five major clades. *Kengyilia changduensis* and *K. tibetica* were placed within the St clade, grouped with most St‐containing species, including diploid *Pseudoroegneria* species with the StSt genome, 40 polyploid species harboring the St subgenome, *Lophopyrum elongatum* (Host) Á. Löve and *L. bessarabicum* (Savul. & Rayss) C.Yen et J. L. Yang with the EE genome, and *Dasypyrum villosum* (L.) Canargy with the VV genome. The Clade A/C/D/K/Q/R/S/T/U contained the *Triticum*‐*Aegilops* complex, *Taeniatherum* (TaTa genome), *Secale* (RR genome), *Crithopsis* (KK genome), and *Heteranthelium* (QQ genome). The third clade (F/O/P/W/Xe) comprised diploid *Agropyron cristatum* and *A. mongolicum* Keng with the PP genome, and the polyploid *K. melanthera* (Keng) J. L. Yang, C. Yen et B. R. Baum and *Kengyilia dingqinensis* (StStYYPP genome). This clade also included *Eremopyrum triticeum* (Gaertn.) Nevski (XeXe genome), *Eremopyrum distans* (C. Koch) Nevski (FF genome), *Australopyrum pectinatum* var. *retrofractum* (J. W. Vickery) C. Yen et J. L. Yang (WW genome), and *Henradia persica* (Boiss.) C. E. Hunnard (OO genome). The remaining two clades consisted of *Hordeum* (H/I clade, HH and II genomes) and *Psathyrostachys* (Ns clade, NsNs genome). Thus, *Kengyilia melanthera* and *K. dingqinensis* possessed a PP genome maternal donor, whereas other *Kengyilia* species, including *K. changduensis* and *K. tibetica*, which shared the typical St‐type plastome.

## 4 Discussion

### 4.1 The Three Species Should Be Treated as New Taxa in *Kengyilia*

The morphological characteristics of *Kengyilia changduensis*, *K. tibetica*, and *K. dingqinensis* differed from all previously described species in *Kengyilia*. Notably, these species display awned glumes and lemmas with relatively longer awns, and *K. dingqinensis* is further characterized by pubescent glumes. GISH confirmed that all three species are hexaploidy (2*n* = 6*x* = 42), with the genome constitution StStYYPP. According to the genomic classification of Triticeae (Dewey 1984; Wang et al. 1994; Yen et al. 2005), the genome formula places them unequivocally within *Kengyilia*. Phylogenetic analysis of *GBSSI* sequences revealed that each of the three species harbors three distinct haplotypes corresponding to the St, Y, and P subgenomes. Together, the GISH and phylogenetic findings indicated that the St and Y genomes derived from *Roegneria*, while the P genome was donated by *Agropyron*. To sum up, the morphological, cytogenetic, and molecular evidence supports the recognition of these three entities as new species belonging to the genus *Kengyilia*.

### 4.2 Taxonomic Treatment of the Three New Species in *Kengyilia*

**FIGURE 6:**
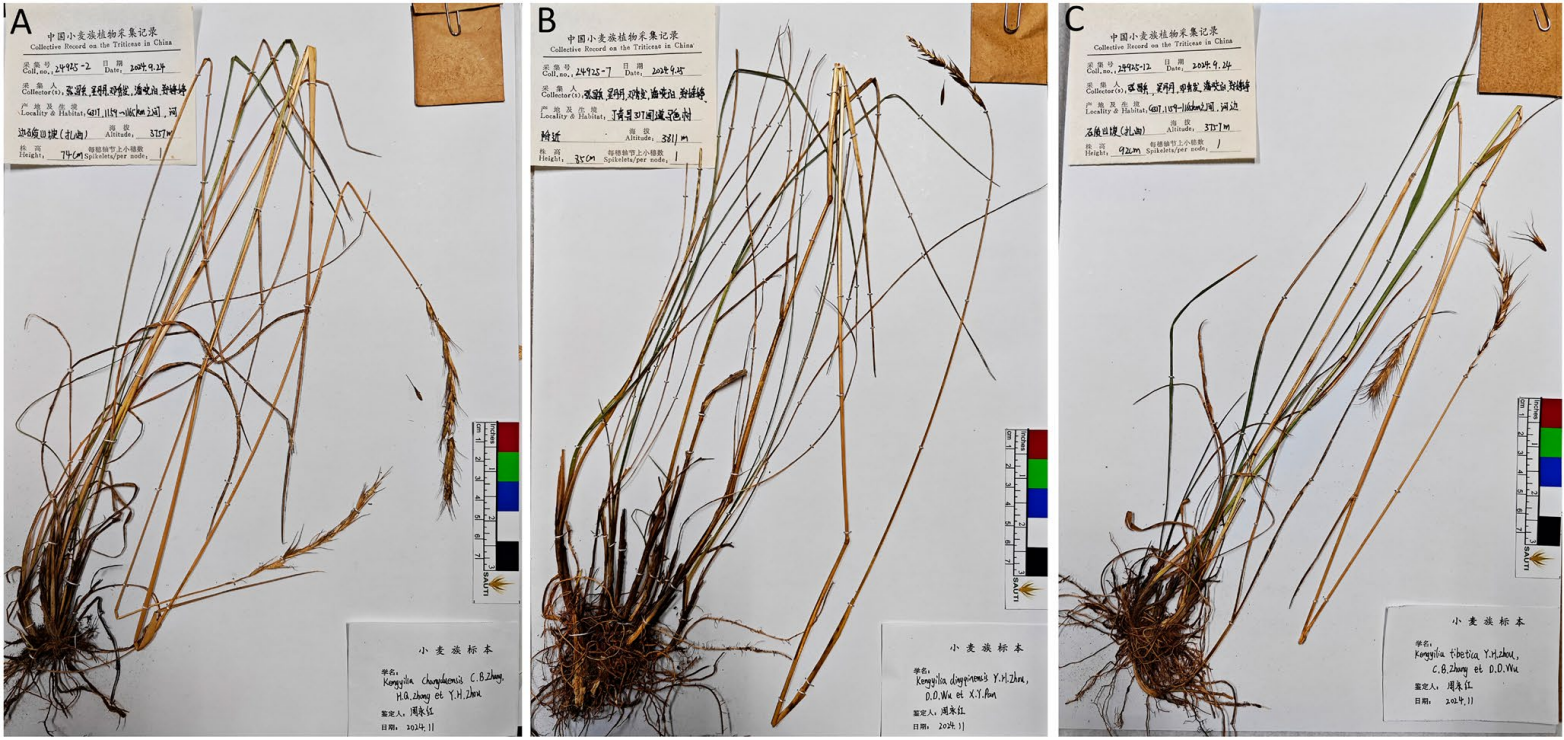
The holotype of the three new *Kengyilia* species. (A) *Kengyilia changduensis* C. B. Zhang, H. Q. Zhang et Y. H. Zhou; (B) *Kengyilia dingqinensis* Y. H. Zhou, D. D. Wu et X. Y. Pan; (C) *Kengyilia tibetica* Y. H. Zhou, C. B. Zhang et D. D. Wu.

***Kengyilia changduensis*** C. B. Zhang, H. Q. Zhang et Y. H. Zhou, sp. nov. Figures 2A, 6A **昌都仲彬草**.

**Type**: CHINA, Tibet, Changdu, Leiwuqi, Rebuzitang Village, in the stony slope of Zhaqu River, 3757 m, 24 September 2024, C. B. Zhang, D. D. Wu, X. F. Deng, X. Y. Pan & T. T. Zheng (collection number: 24925‐2; holotype: SAUTI; isotype: SAUTI).

**Diagnosis**: *Kengyilia changduensis* is morphologically the most similar species to *Kengyilia laxiflora* (Keng) J. L. Yang, C. Yen et B. R. Baum, but it is distinguished from *K. laxiflora* by its awn of glume (2 mm vs. absent) and longer awns of lemmas (5–10 mm vs. 1–2 mm).

**Description**: Perennial herb, cespitose; culms usually erect, 70–110 cm tall. Basal leaf sheaths with sparse hairs; blades flat, 10–22 cm long, 2–5 mm wide, glabrous. Spikes slightly bending, sparse, purplish at maturity, 8–12 cm long (excluding awns), 6–8 mm wide, and 10–15 spikelets per spike; 1 spikelet per node; internodes 3–15 mm long. Spikelets 15–25 mm (excluding awns) long, with 6–9 florets. Glumes nearly equal length, oblong‐lanceolate, margin membranous, 4–6 mm long (excluding awns), 3–5 prominent veins, awned, awns 2 mm. Lemmas oblong, pubescent, 5 veins, first lemma 5 mm, awned, awns 5–10 mm. Paleas oblong, equal length nearly equal with lemma, apex concave. Chromosome number 2*n* = 6*x* = 42; genome constitution StStYYPP.

**Phenology**: Flowering: June–July; Fruiting: September–October.

**Distribution and habitat**: *Kengyilia changduensis* is known only from a few localities along the Zhaqu River valley of Changdu, Tibet, China. It was found among bushes in river valleys or on stony mountain slopes between 3500 and 3800 m, growing together with *Elymus sibiricus* L., *Roegneria laxinodis* L. B. Cai, *Roegneria retroflexa* (B. R. Lu et B. Salmon) L. B. Cai, *Campeiostachys breviaristata* (Keng) Y. H. Zhou, H. Q. Zhang et C. R. Yang, *Campeiostachys nutans* (Griseb.) J. L. Yang B. R. Baum et C. Yen, and *Campeiostachys dahurica* (Turcz. ex Griseb.) J. L. Yang, B. R. Baum et Yen var. *cylindrica* (Franch.) J. L. Yang, B. R. Baum et C. Yen.

***Kengyilia dingqinensis*** Y. H. Zhou, D. D. Wu et X. Y. Pan, sp. nov. Figures 2B, 6B **丁青仲彬草**.

**Type**: CHINA, Tibet, Changdu, Dingqin, Luorongka Village, in the stony slope of Sequ River, 3811 m, 25 September 2024, C. B. Zhang, D. D. Wu, X. F. Deng, X. Y. Pan & T. T. Zheng (collection number: 24925‐7; holotype: SAUTI; isotype: SAUTI).

**Diagnosis**: *K. dingqinensis* is morphologically the most similar species to *Kengyilia hirsuta* (Keng) J. L. Yang, C. Yen et B. R. Baum, but it is distinguished from *K. hirsuta* by its arrange of loose spikelets (loose vs. tight), awn of glume (2 mm vs. absent) and covered with soft hairs (long and soft hairs vs. absent), and lemmas covered with soft hairs (soft hairs vs. hard hairs) and longer awns (5 mm vs. 2.5–6 mm).

**Description**: Perennial herb, cespitose; culms usually erect, 70–100 cm tall. Basal leaf sheaths with sparse hairs; blades flat, 20–32 cm long, 2–4 mm wide, glabrous. Spikes slightly bending, 7–12 cm long (excluding awns), lower part of the spikes is sparse, internodes hairy, 5–6 mm wide, and 7–12 spikelets per spike; 1 spikelet per node; rachises scabrous and margin shortly barbellulate; internodes 3–20 mm long. Spikelets 15–25 mm (excluding awns) long, with 3–5 florets. Glumes nearly equal length, covered with soft hairs, oblong, margin membranous, 4–6 mm long (excluding awns), 3–5 prominent veins, awned, awns 2 mm. Lemmas oblong, covered with soft hairs, 5 veins, first lemma 4–6 mm, awned, awns 5 mm. Paleas oblong, equal length with lemma, apex concave. Chromosome number 2*n* = 6*x* = 42; genome constitution StStYYPP.

**Phenology**: Flowering: June–July; Fruiting: September–October.

**Distribution and habitat**: *Kengyilia dingqinensis* is known only from a few localities along the Sequ River valley of Changdu, Tibet, China. It was found among bushes in river valleys or on stony mountain slopes between 3700 and 3900 m, growing together with *Elymus sibiricus* L., *Roegneria stricta* Keng, *Campeiostachys breviaristata* (Keng) Y. H. Zhou, H. Q. Zhang et C. R. Yang and *Campeiostachys nutans* (Griseb.) J. L. Yang B. R. Baum et C. Yen.

***Kengyilia tibetica*** Y. H. Zhou, C. B. Zhang et D. D. Wu, sp. nov. Figures 2C, 6C **西藏仲彬草**.

**Type**: CHINA, Tibet, Changdu, Karuo, Mipava Village, in the stony slope of Zhaqu River, 3570 m, 24 September 2024, C. B. Zhang, D. D. Wu, X. F. Deng, X. Y. Pan & T. T. Zheng (collection number: 24925‐12; holotype: SAUTI; isotype: SAUTI).

**Diagnosis**: *K. tibetica* is morphologically the most similar species to *Kengyilia laxiflora* (Keng) J. L. Yang, C. Yen et B. R. Baum, but it is distinguished from *Kengyilia laxiflora* by its hairy internodes (hairy vs. smooth), awn of glume (2–6 mm vs. absent), and longer awns of lemmas (10–15 mm vs. 1–2 mm).

**Description**: Perennial herb, cespitose; culms usually erect, 74–110 cm tall. Basal leaf sheaths have sparse hairs; blades flat, 7–16 cm long, 1–3 mm wide, glabrous. Spikes slightly bending, 10–14 cm long (excluding awns), 7–9 mm wide, and 9–15 spikelets per spike; 1 spikelet per node; rachises scabrous and margin shortly barbellulate; internodes hairy, 14–25 mm long. Spikelets 20–25 mm (excluding awns) long, with 5–7 florets. Glumes nearly equal length, oblong‐lanceolate, 3–5 mm long (excluding awns), 3–5 prominent veins, awned, awns 2–6 mm. Lemmas oblong, pubescent, 5 veins, first lemma 4–5 mm, awned, awns 10–15 mm. Paleas oblong, equal length nearly equal with lemma, apex concave. Chromosome number 2*n* = 6*x* = 42; genome constitution StStYYPP.

**Phenology**: Flowering: June–July; Fruiting: September–October.

**Distribution and habitat**: *Kengyilia tibetica* is known only from a few localities along the Zhaqu River valley of Changdu, Tibet, China. It was found among bushes in river valleys or on stony mountain slopes between 3500 and 3600 m, growing together with *Elymus sibiricus* L., *Roegneria breviglumis* Keng, *Roegneria brevipes* Keng, *Roegneria laxinodis* L. B. Cai, *Roegneria retroflexa* (B. R. Lu et B. Salmon) L. B. Cai, *Campeiostachys breviaristata* (Keng) Y. H. Zhou, H. Q. Zhang et C. R. Yang, *Campeiostachys nutans* (Griseb.) J. L. Yang B. R. Baum et C. Yen, and *Campeiostachys dahurica* (Turcz. ex Griseb.) J. L. Yang, B. R. Baum et C. Yen var. *cylindrica* (Franch.) J. L. Yang, B. R. Baum et C. Yen.

### 4.3 Phylogenetic Relationships Among the Three New *Kengyilia* Species and Related Species

Based on morphological characteristics, *Kengyilia changduensis* and *K. tibetica* are similar to *K. laxiflora* , while *K. dingqinensis* resembles *K. hirsuta* . All three new species occur sympatrically in Changdu, Tibet. analyses of nuclear *GBSSI* phylogenies show that the St and P haplotypes of the three species are genetically similar, whereas the Y haplotypes are differentiated. Chloroplast genome analyses place *K. changduensis* and *K. tibetica*, along with related St‐containing polyploid species, comprises multiple genera, such as *Elymus* sensu stricto (StStHH), *Roegneria* C. Koch (StStYY and StStStStYY), *Douglasdeweya* C. Yen, J. L. Yang et B.R. Baum (StStPP), *Trichopyrum* Á. Löve (StStEE, StStEEEE, and StStStStEEEEEE), *Campeiostachys* Drob. (StStYYHH), *Kengyilia* C. Yen et J. L. Yang (StStYYPP), and *Anthosachne* Steudel (StStYYWW), according to the genomic system of classification (Yen et al. 2005). However, Zhang et al. (2009) reported that *Agropyron* served as the maternal parent of *Kengyilia melanthera*, *K. mutica*, and *K. thoroldiana* based on plastid *trn*L‐F segment. In our whole‐chloroplast genome phylogeny, only *K. melanthera* and the new species *K. dingqinensis* group with *Agropyron*. This discrepancy likely arises because single or few fragments, especially rapidly evolving intergenic spacers, may be influenced by unsuitable evolving rate, which will obscure deep evolutionary relationships (Clegg et al. 1994; Chen, Yan, et al. 2021). Notably, the phylogenetic clustering of the three new species is congruent with their morphological affinities: *K. changduensis* and *K. tibetica* are both morphological and phylogenetical close, whereas *K. dingqinensis* is more divergent.

At present, we found no clear evidence that differences in maternal origin are directly associated with the observed morphological variation among the three species. Although *K. changduensis* and *K. tibetica* appear to have originated from a *Roegneria*‐like maternal lineage, whereas *K. dingqinensis* is more closely associated with an *Agropyron*‐like maternal lineage, these differences are not reflected by obvious diagnostic morphological characters. A possible explanation is the strong influence of the St genome, which is shared by all three species. Previous studies have shown that St‐containing taxa often exhibit substantial morphological similarity across species and even across genera, suggesting that the conserved genomic background may mask phenotypic signals associated with different parental origins (Fan et al. 2013). Although *K. changduensis* and *K. tibetica* are morphologically and phylogenetically similar, they exhibit stable morphological differences—including awn length and internode pubescence—under both wild and cultivated conditions. Therefore, we consider these differences to be interspecific. To further evaluate the degree of differentiation among *K. changduensis* and *K. tibetica*, future studies should generate F_1_ hybrids between them and their morphologically similar relatives and examine meiotic chromosome pairing.

Regarding the degree of genetic divergence, our phylogenetic analyses recovered the three taxa as distinct evolutionary lineages rather than as populations nested within a single species, suggesting that the observed divergence is more consistent with interspecific differentiation than with ordinary intraspecific variation. Therefore, *K. changduensis* and *K. tibetica* are genetically closely related and the tetraploid species of *Roegneria* served as the maternal donors, whereas *K. dingqinensis* followed a distinct evolutionary pathway, with *Agropyron* as the maternal donor.

## 5 Conclusion

Based on morphological observation and statistical analysis, GISH, and phylogenetic analysis of DNA regions, we conclude that *Kengyilia changduensis*, *K. dingqinensis*, and *K. tibetica* are three new *Kengyilia* species with the StStYYPP genomic constitution. The phylogenetic relationship of *GBSSI* and the chloroplast genome of these three new *Kengyilia* species and other *Kengyilia* has a close relationship, and is also closely related to *Roegneria* and *Agropyron*. The maternal origin of *Kengyilia dingqinensis* and *K. melanthera* is the PP genome of diploid species in *Agropyron*, which differs from other *Kengyilia* species.

## Author Contributions

**Xiao‐Yang Pan:** methodology (equal), resources (equal), software (equal), writing – original draft (equal). **Shuang‐Dan Li:** methodology (equal). **Chang‐Bing Zhang:** resources (equal). **Jia‐Yue He:** methodology (equal). **Yuan‐Yuan Lu:** methodology (equal). **Ting‐Ting Zheng:** methodology (equal). **Ying‐Hui Li:** data curation (equal), funding acquisition (equal), writing – review and editing (supporting). **Li‐Li Xu:** funding acquisition (equal). **Hai‐Qin Zhang:** methodology (equal), resources (equal), writing – review and editing (supporting). **Hou‐Yang Kang:** funding acquisition (equal), resources (equal), supervision (equal). **Dan‐Dan Wu:** conceptualization (equal), funding acquisition (equal), supervision (equal), writing – review and editing (equal). **Yong‐Hong Zhou:** conceptualization (lead), formal analysis (equal), methodology (equal), resources (equal), supervision (equal), writing – review and editing (lead).

## Funding

This work was supported by the Science and Technology Department of Sichuan Province (2026NSFSC0206), National Key Research and Development Program of China (2024YFD1201202), National Science and Technology Major Project (NK20220607).

## Conflicts of Interest

The authors declare no conflicts of interest.

## Supporting information


**Figure S1:** Gene map of the *K. tibetica* chloroplast genome. Genes inside and outside of the circle are transcribed in the clockwise and counterclockwise directions, respectively. The colored bars indicate known protein‐coding genes, tRNA genes and rRNA genes.

**Table S1:** Primers used in this study.
**Table S2:** Thermocycling conditions for amplification of genes using the PCR.
**Table S3:** Materials used in this study.

## Data Availability

The data sets supporting the results of this article were deposited in the GenBank (https://www.ncbi.nlm.nih.gov/) and GenBase (https://ngdc.cncb.ac.cn/genbase/) repository. We have added the accession numbers of sequences obtained in this study within the article and its Table S3.
