# Supplementary material for: Biosystematics and Taxonomic Treatment of Three New Kengyilia Species in the Triticeae (Poaceae)
Source: Ecol Evol. 2026 Jul 23;16(7):e74040. doi: 10.1002/ece3.74040 (PMC13395575; doi:10.1002/ece3.74040)
Supplement: Supplementary file 1 — Figure S1: Gene map of the K. tibetica chloroplast genome. Genes inside and outside of the circle are transcribed in the clockwise and counterclockwise directions, respectively. The colored bars indicate known protein‐coding genes, tRNA genes and rRNA genes. [file ECE3-16-e74040-s002.docx]

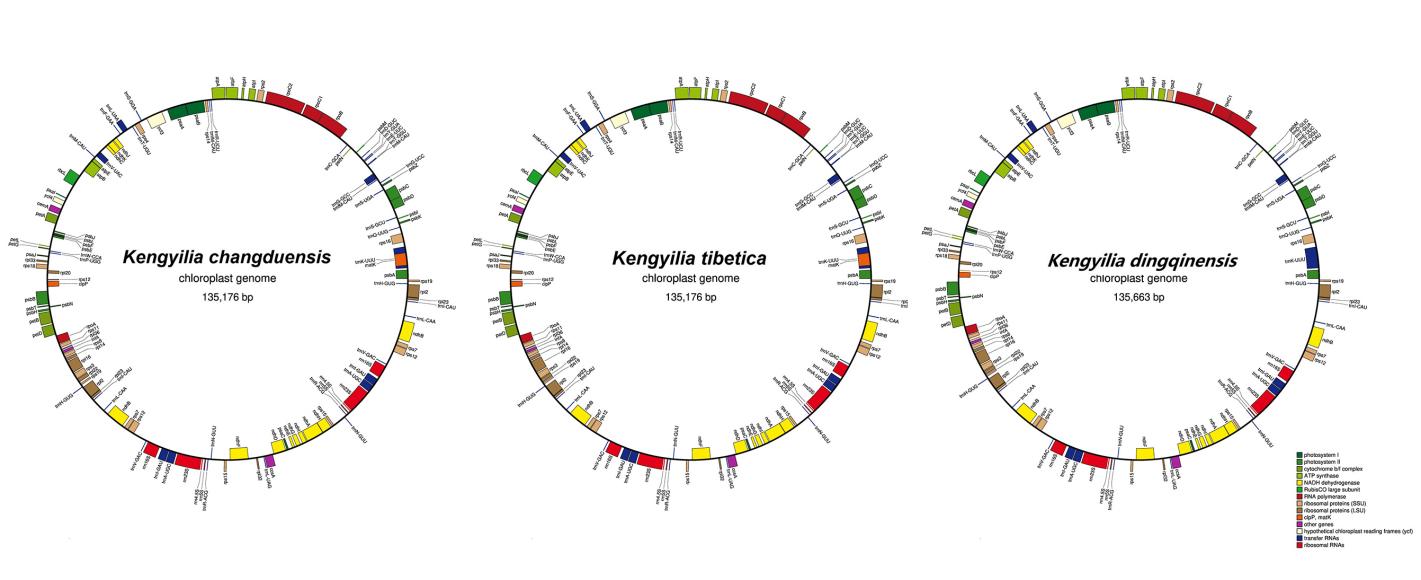


**Fig. S1** Circular map of the chloroplast genome of the *Kengyilia changduensis*, *K. tibetica,* and *K. dingqinensis*. Genes shown inside the circle are transcribed clockwise, whereas those outside the circle are transcribed counterclockwise. Genes belonging to different functional groups are indicated by different colors.
